# Whole exome sequencing identifies multiple novel candidate genes in familial gastroschisis

**DOI:** 10.1002/mgg3.1176

**Published:** 2020-03-12

**Authors:** Víctor M. Salinas‐Torres, Hugo L. Gallardo‐Blanco, Rafael A. Salinas‐Torres, Ricardo M. Cerda‐Flores, José J. Lugo‐Trampe, Daniel Z. Villarreal‐Martínez, Marisol Ibarra‐Ramírez, Laura E. Martínez de Villarreal

**Affiliations:** ^1^ Department of Genetics School of Medicine and University Hospital Dr. José Eleuterio González Universidad Autónoma de Nuevo León Monterrey México; ^2^ Department of Systems and Computing Instituto Tecnológico de Tijuana Tijuana México; ^3^ School of Nursing Universidad Autónoma de Nuevo León Monterrey México

**Keywords:** abdominal wall defect, alleles, development, gastroschisis, genes, genetics, next‐generation sequencing, pathogenesis, recurrence, whole exome sequencing

## Abstract

**Background:**

Genetic association studies for gastroschisis have highlighted several candidate variants. However, genetic basis in gastroschisis from noninvestigated heritable factors could provide new insights into the human biology for this birth defect. We aim to identify novel gastroschisis susceptibility variants by employing whole exome sequencing (WES) in a Mexican family with recurrence of gastroschisis.

**Methods:**

We employed WES in two affected half‐sisters with gastroschisis, mother, and father of the proband. Additionally, functional bioinformatics analysis was based on SVS–PhoRank and Ensembl–Variant Effect Predictor. The latter assessed the potentially deleterious effects (high, moderate, low, or modifier impact) from exome variants based on SIFT, PolyPhen, dbNSFP, Condel, LoFtool, MaxEntScan, and BLOSUM62 algorithms. The analysis was based on the Human Genome annotation, GRCh37/hg19. Candidate genes were prioritized and manually curated based on significant phenotypic relevance (SVS–PhoRank) and functional properties (Ensembl–Variant Effect Predictor). Functional enrichment analysis was performed using ToppGene Suite, including a manual curation of significant Gene Ontology (GO) biological processes from functional similarity analysis of candidate genes.

**Results:**

No single gene‐disrupting variant was identified. Instead, 428 heterozygous variations were identified for which *SPATA17, PDE4DIP, CFAP65, ALPP, ZNF717, OR4C3, MAP2K3, TLR8,* and* UBE2NL* were predicted as high impact in both cases, mother, and father of the proband. *PLOD1, COL6A3, FGFRL1, HHIP*,* SGCD*,* RAPGEF1, PKD1*,* ZFHX3*,* BCAS3*,* EVPL*,* CEACAM5,* and* KLK14* were segregated among both cases and mother. Multiple interacting background modifiers may regulate gastroschisis susceptibility. These candidate genes highlight a role for development of blood vessel, circulatory system, muscle structure, epithelium, and epidermis, regulation of cell junction assembly, biological/cell adhesion, detection/response to endogenous stimulus, regulation of cytokine biosynthetic process, response to growth factor, postreplication repair/protein K63‐linked ubiquitination, protein‐containing complex assembly, and regulation of transcription DNA‐templated.

**Conclusion:**

Considering the likely gene‐disrupting prediction results and similar biological pattern of mechanisms, we propose a joint “multifactorial model” in gastroschisis pathogenesis.

## Graphical abstract

Heritable factors for gastroschisis have remained unexplained as the low‐frequency and rare variants have not yet been explored. Technological platforms such as NGS and WES could help in the understanding of the biological basis for gastroschisis. We identified multiple novel susceptibility genes involved in crucial GO biological processes possibly underlying gastroschisis. A joint “multifactorial model” is proposed for gastroschisis that might impact the normal ventral body wall closure as well as gastroschisis developmental process.

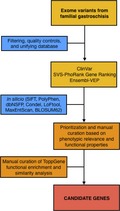

## 1 INTRODUCTION

Gastroschisis is a birth defect characterized by viscera protruding without a covering sac, which involves the ventral body wall development and the umbilical ring. The increasing prevalence of the disease along with its etiology is poorly understood (Salinas‐Torres, Salinas‐Torres, Cerda‐Flores, & Martínez‐de‐Villarreal, 2018a). Studies suggest that gastroschisis pathogenesis is likely a result of aberrant angiogenesis, cell–cell interaction, or inflammatory response toward environmental factors combined with individuals’ genetic susceptibility (Salinas‐Torres, Salinas‐Torres, Cerda‐Flores, Gallardo‐Blanco, & Martínez‐de‐Villarreal, 2018; Salinas‐Torres, Salinas‐Torres, Cerda‐Flores, & Martínez‐de‐Villarreal, 2018b). An estimated overall recurrence risk of 5.7% from population‐based studies suggests that genetic factors may account for gastroschisis susceptibility. Among familial gastroschisis, higher risk in patient's siblings than in parents was reported (Salinas‐Torres, Salinas‐Torres, Cerda‐Flores, & Martínez‐de‐Villarreal, 2018c).

Linkage analysis and genetic association studies are the most common approaches to identify variants linked to a disease. Yet, family‐based studies represent the preferred approach to decipher underlying genetic factors segregating between affected relatives, allowing better detection of novel susceptibility variants than the screening of pooled unrelated cases and controls. Heritable factors of gastroschisis have remained unexplained as the low frequency and rare variants have not yet been explored using technological platforms such as next‐generation sequencing (NGS) and whole exome sequencing (WES). These technologies could help in understanding the biological basis of gastroschisis by exploring the functional consequences of genomic variation and addressing new insights into human biology for gastroschisis (Chakravorty & Hegde, 2018).

To explore these genetic factors implicated in gastroschisis, we employ WES in a Mexican family with recurrence of gastroschisis in order to identify candidate genes and their genetic variations that may be associated with gastroschisis risk.

## 2 MATERIALS AND METHODS

### 2.1 Study design and participants

The present study was conducted according to the guidelines for good clinical practices and was approved by the Institutional Ethics Committee from the School of Medicine and University Hospital Dr. José Eleuterio González, Universidad Autónoma de Nuevo León, México (Approval: GN17‐00002). A Mexican family with recurrence of gastroschisis was assessed for WES based on the presence of at least two related first‐ or second‐degree gastroschisis cases, regardless of their age, and without evidence of multiple congenital anomalies or a syndrome. Written informed consents, comprehensive clinical histories, and blood samples were collected and sampled from the family members (two affected half‐sisters, mother, and father of the proband) in the Genetics Department of our University.

### 2.2 Nucleic acid extraction

For each participant, total genomic DNA was extracted using the QIAamp^®^ DNA Blood Mini kit and the automated QIAcube system (cat no. 158445; Qiagen GmbH, Hilden, Germany) following the recommended protocol. Purified DNA was collected and stored at ‑20°C before analysis.

### 2.3 Whole exome sequencing and data analysis

**Figure 1:**
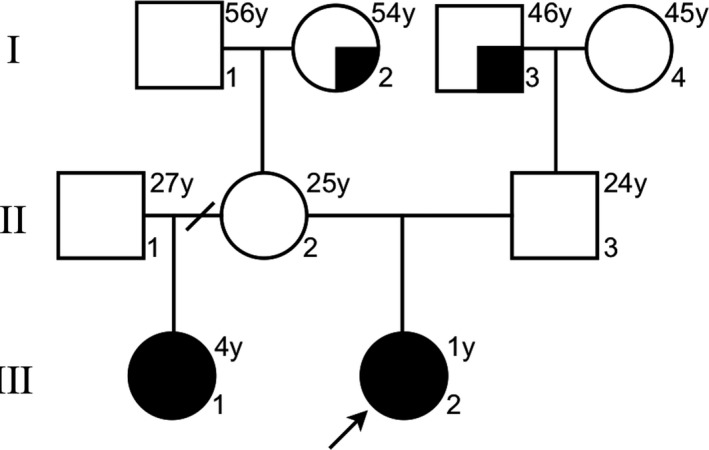
Pedigree of the Mexican family with recurrence of gastroschisis. Square and circle symbols depict males and females, respectively. Solid symbols indicate gastroschisis cases (III‐1 and III‐2), semisolid symbols indicate cases of umbilical hernia (I‐2 and I‐3), and the open symbols indicate unaffected individuals. The arrow points to the proband

The investigated family has two affected half‐sisters with different father, same mother (the parents were unaffected; Figure 1). Initially, Centogene AG^®^ performed a trio‐based WES for the affected index patient and her parents. Fragmented genomic DNA was subject to library construction. Exome capture was performed using Nextera^®^ Rapid Capture Exome, Illumina^®^ and the generated library sequenced on an Illumina^®^ platform to an average coverage depth 100‐130X. Subsequently, LC Sciences^®^ performed the WES for the affected half‐sister (her father was not assessed). Fragmented genomic DNA was subject to library construction. Exome capture was performed using SureSelect^®^ Human All Exon V6, Agilent^®^ following the recommended protocol and sequencing was performed using the Illumina^®^ Hiseq X Ten at LC‐BIO for a 150‐bp paired‐end run. An end‐to‐end from each in‐house bioinformatics pipelines including base calling, first filtering of low‐quality reads, duplicates, and probable artifacts, alignment, quality controls, and annotation of variants were applied (Centogene AG^®^ and LC Sciences^®^). Variants related to the phenotype were reported and in‐house validated to exclude NGS artifacts.

Exome DNA sequences were mapped to their location in the build of the Human Genome annotation (GRCh37/hg19). Single‐nucleotide variants (SNVs) and insertions and deletions of nucleotides (INDELs) annotated were filtered according to variants identified as intronic (intron boundaries ± 20 bp), noncoding, or synonymous, as well as variants with an allele frequency >1% either in ExAc database (http://exac.broadinstitute.org) or 1,000 Genomes (http://www.internationalgenome.org), meanwhile all relevant inheritance patterns and disease‐causing variants reported in HGMD^®^ (http://www.hgmd.cf.ac.uk/), ClinVar (https://www.ncbi.nlm.nih.gov/clinvar/), CentoMD^®^ (https://www.centomd.com/), Genome (ftp://ftp.ensembl.org/pub/release-73/fasta/homo_sapiens/dna/Homo_sapiens.GRCh37.73.dna.toplevel.fa.gz), and dbSNP (https://www.ncbi.nlm.nih.gov/projects/SNP/) were considered.

Once the sample information from the two sets of reports by CENTOGENE AG^®^ and LC Sciences^®^ was obtained, unifying database was set up in order to have a better visualization of the SNVs and INDELs annotated and considered (Salinas‐Torres, Gallardo‐Blanco, Salinas‐Torres, & Martínez de Villarreal, 2019a,b).

### 2.4 Functional bioinformatic analysis

Exome variants were filtered and prioritized against their phenotypic relevance (“Gastroschisis”) based in SNP & Variation Suite (2018); (SVS v8.8.1)–PhoRank gene ranking (Golden Helix^®^, Inc.; http://wwwgoldenhelix.com). Also, Ensembl–VEP bioinformatic platform was used to assess the potential deleterious effects (high, moderate, low, and modifier impact) of the exome variants based on a combined effect of the following algorithms: Sorting Tolerant From Intolerant (SIFT), Polymorphism Phenotyping (PolyPhen), database for Non‐Synonymous single‐nucleotide variant and Functional Prediction (dbNSFP), CONsensus DELeteriousness score of missense single nucleotide variant (Condel), Loss‐of‐Function mutations (LoFtool), MaxEntScan (splicing prediction), and BLOSUM62 (conservation prediction; Zerbino et al., 2018). The analysis was based on the Human Genome annotation, GRCh37/hg19.

**Figure 2:**
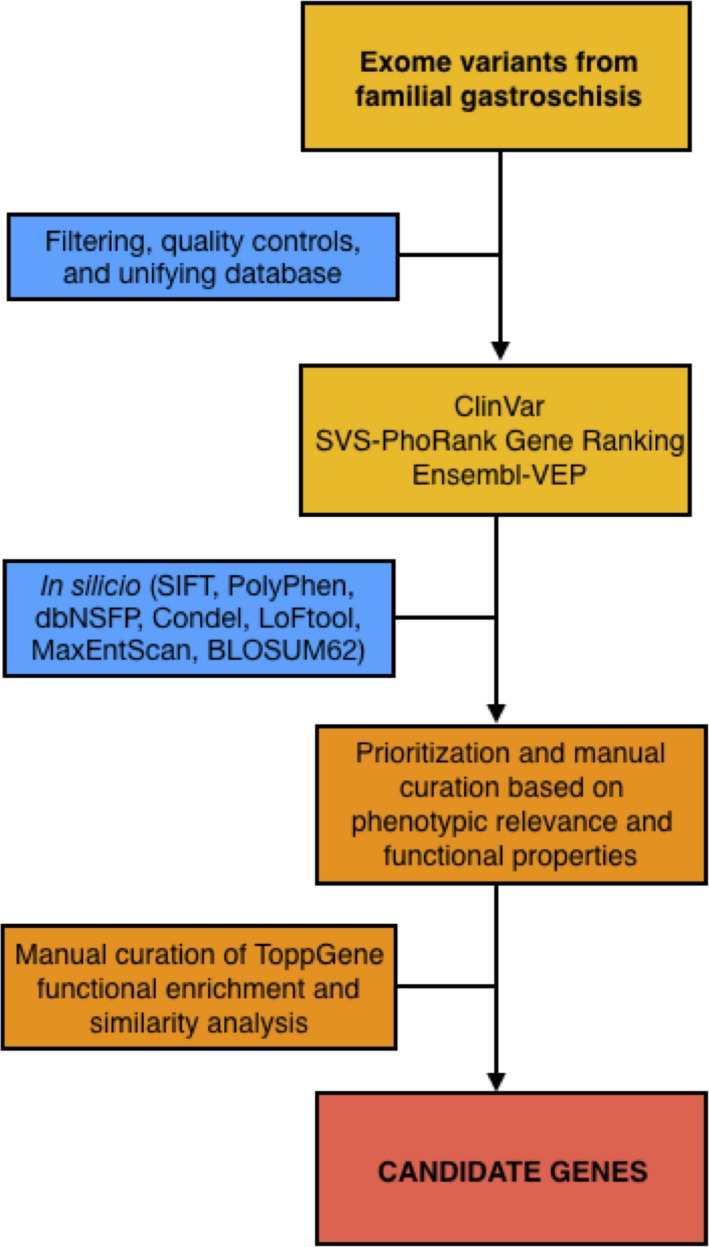
Workflow for the identification of candidate genes from familial gastroschisis. Gene variants were prioritized based on phenotypic relevance and functional properties by independent bioinformatics platforms and in silico analysis (predicted deleterious effect as stop gained, start and stop lost, nonsense‐mediated decay transcript variant, splice region variant, or missense variants)

Candidate genes were prioritized and manually curated based on significant phenotypic relevance according to SVS–PhoRank gene ranking (Golden Helix^®^, Inc.; http://wwwgoldenhelix.com), functional properties according to Ensembl–VEP (high impact; Zerbino et al., 2018), and those segregating among both affected half‐sisters and the mother. Functional enrichment analysis was performed using ToppGene Suite (Chen, Bardes, Aronow, & Jegga, 2009), including a manual curation of significant Gene Ontology (GO) biological processes from functional similarity analysis of candidate genes (Figure 2). GO terms were selected based on their proximity and plausibility to the phenotype (Salinas‐Torres, et al., 2018; Salinas‐Torres et al., 2018b).

## 3 RESULTS

### 3.1 Familial clinical data

The family pedigree is illustrated in Figure 1. The proband, a 1‐year‐old female, was born at the 37th week of gestation to a 24‐year‐old G2 C1 woman (prepregnancy body mass index was 19.2) and her 24‐year‐old husband. Delivery was by cesarean section after prenatal diagnosis of gastroschisis. Apgar score was 9, birth weight 2,050 g (<3rd centile), and length 48 cm (15–50th centile). Her half‐sister, a 4‐year‐old child, was born at the 36th week of gestation. At birth, the mother was 21‐year‐old (prepregnancy body mass index was 18.4) and her former husband was 23‐year‐old (not whole exome sequenced). Delivery was by cesarean section after prenatal diagnosis of gastroschisis. Apgar score was 9, birth weight 1,670 g, and length 42 cm (both were <3rd centile). Consanguinity was ruled out. Postnatally, in both cases, extrusion of dilated small and large intestinal loops through the open right side of the umbilical ring was observed. Gastroschisis was confirmed as an isolated anomaly and a primary closure of the abdominal wall closure was performed without further clinical complications. Placenta and membranes were normal.

Tobacco smoking (1–2 cigarettes per day) and alcohol consumption (1–2 beer cans of 355 ml per week) at preconception and during the first trimester of pregnancy were detected on parental clinical histories. The mother referred genitourinary infections during the second trimester of pregnancy in both cases as well as a limited preconceptional care (less than four clinical follow‐ups in each pregnancy). There was no consumption of folic acid‐containing supplements.

### 3.2 Screening, identification, and bioinformatic analysis of novel candidate variants

No pathological DNA variants associated with gastroschisis were detected by CENTOGENE AG^®^ and LC Sciences^®^. A total of 17,354 variants of the two sets of reports provided by CENTOGENE AG^®^ (3,518 variants) and LC Sciences^®^ (13,836 variants) were considered (Salinas‐Torres et al., 2019a,b). As to 31 August 2018, according to ClinVar (https://www.ncbi.nlm.nih.gov/clinvar/), SVS–PhoRank gene ranking (http://wwwgoldenhelix.com), and Ensembl–VEP (Zerbino et al., 2018), a total of 428 heterozygous DNA variations have been prioritized and manually curated by phenotypic relevance and functional properties (Salinas‐Torres et al., 2019a,b).

Thus, a combined effect of in silico prediction tools identified 10 high‐impact potential deleterious variants in both cases, mother, and father of the proband (Table 1), whereas 12 genetic variants segregating among both cases with gastroschisis and the mother were identified (Table 2). Multiple moderate and modifier impact variants segregating within the family were also identified (Salinas‐Torres et al., 2019a,b). Of note, the following variants were also identified as modifier impact variants in addition to being predicted as having a high or moderate deleterious effect: *SPATA17* (rs147297747), *PDE4DIP* (rs61804988), *CFAP65* (rs6736922), *ALPP* (rs13026692), *ZNF717* (rs150689808 and rs140982339), *MAP2K3* (rs55796947), *TLR8* (rs3764880), *COL6A3* (rs202092407)*, FGFRL1* (rs4647931), *RAPGEF1* (rs554713394), *PKD1* (rs142888788), *ZFHX3* (rs200992486), *BCAS3* (rs201061353), *EVPL* (rs202057945), *CEACAM5* (rs998112305).

**Table 1:** High‐impact genetic variants identified in both cases with gastroschisis, mother, and father of the proband[Link]

Chr	Genes	Variants	HGVS nomenclature	Consequence details	MAF (*n*)
1	*SPATA17*	rs147297747	ENST00000470448.5:c.2T>C, ENSP00000473514.1:p.Met1?	Start lost, NMDTV	1.997E‐04 (1)[Link]
	*PDE4DIP*	rs61804988	ENST00000369356.8:c.7053G>A, ENSP00000358363.4:p.Trp2351Ter	Stop gained	0.500 (122,829)[Link]
2	*CFAP65*	rs6736922	ENST00000436631.5:c.1A>C, ENSP00000396836.1:p.Met1?	Start lost	4.073E‐06 (1)[Link]
	*ALPP*	rs2260309	ENST00000392027.2:c.804T>G, ENSP00000375881.2:p.Tyr268Ter	Stop gained	0.617 (141,172)[Link]
3	*ZNF717*	rs150689808	ENST00000478296.5:c.596C>G, ENSP00000419377.1:p.Ser199Ter	Stop gained	7.964E‐06 (1)[Link]
		rs140982339	ENST00000468296.5:c.107G>A, ENSP00000418187.1:p.Trp36Ter	Stop gained	0.021 (958)[Link]
11	*OR4C3*	rs72473368	ENST00000319856.5:c.441G>A, ENSP00000321419.5:p.Trp147Ter	Stop gained	0.500 (120,864)[Link]
17	*MAP2K3*	rs55796947	ENST00000496046.5:c.304C>T, ENSP00000464043.1:p.Gln102Ter	Stop gained, NMDTV	0.500 (119,837)[Link]
X	*TLR8*	rs3764880	ENST00000218032.6:c.1A>G, ENSP00000218032.6:p.Met1?	Start lost, SRV	0.464 (1751)[Link]
	*UBE2NL*	rs237520	ENST00000618570.1:c.266G>T, ENSP00000488314.1:p.Ter89LeuextTer?	Stop lost	0.617 (2,331)[Link]

Exome variants as stop gained variant, start and stop lost variant, NMDTV, Nonsense‐mediated decay transcript variant; SRV, Splice region variant, with combined effect by functional prediction tools (Zerbino et al., 2018); HGVS, Human Genome Variation Society Nomenclature; MAF, Minor allelic frequency from all individual populations.

1000 genomes project phase 3.

gnomAD exomes 9.

Trans‐Omics for Precision Medicine program.

**Table 2:** Genetic variants segregating among both cases with gastroschisis and the mother[Link]

Chr	Genes	Variants	HGVS nomenclature	Consequence details	MAF (*n*)
1	*PLOD1*	rs201661871	ENST00000376369.3:c.1612‐8C>T	SRV	1.997E‐04 (1)[Link]
2	*COL6A3*	rs202092407	ENST00000295550.4:c.7007C>T, ENSP00000295550.4:p.Pro2336Leu	Missense variant	3.993E‐04 (2)[Link]
4	*FGFRL1*	rs4647931	ENST00000264748.6:c.1271G>T, ENSP00000264748.6:p.Arg424Leu	Missense variant	0.007 (36)[Link]
	*HHIP*	rs753937179	ENST00000296575.3:c.2044C>A, ENSP00000296575.3:p.Leu682Ile	Missense variant	1.018E‐04 (25)[Link]
5	*SGCD*	rs727503423	ENST00000337851.4:c.494G>A, ENSP00000338343.4:p.Arg165Gln	Missense variant	1.236E−04 (30)[Link]
9	*RAPGEF1*	rs554713394	ENST00000372190.3:c.1457C>T, ENSP00000361264.3:p.Thr486Met	Missense variant	2.035E‐05 (5)[Link]
16	*PKD1*	rs142888788	ENST00000423118.5:c.8530G>A, ENSP00000399501.1:p.Val2844Ile	Missense variant, NMDTV	1.997E‐04 (1)[Link]
	*ZFHX3*	rs200992486	ENST00000268489.9:c.10831C>T, ENSP00000493252.1:p.His3611Tyr	Missense variant	0.005 (24)[Link]
17	*BCAS3*	rs201061353	ENST00000390652.9:c.2122G>A, ENSP00000375067.4:p.Asp708Asn	Missense variant	1.950E‐04 (48)[Link]
	*EVPL*	rs202057945	ENST00000586740.1:c.296T>C, ENSP00000465630.1:p.Leu99Pro	Missense variant	2.321E‐04 (57)[Link]
19	*KLK14*	rs112658494	ENST00000391802.1:c.412C>T, ENSP00000375678.1:p.Arg138Trp	Missense variant	0.004 (21)[Link]
	*CEACAM5*	rs998112305	ENST00000435837.2:c.64+12306G>A, ENSP00000385072.1:p.Gly651Arg	Missense variant	8.121E‐06 (2)[Link]

Exome variants as missense, NMDTV, Nonsense‐mediated decay transcript variant; SRV, Splice region variant, with combined effect by functional prediction tools (Zerbino et al., 2018); HGVS, Human Genome Variation Society Nomenclature; MAF, Minor allelic frequency from all individual populations.

1000 genomes project phase 3.

gnomAD exomes 9.

Manual curation of functional enrichment and similarity analyses according to ToppGene Suite (Chen et al., 2009) identified the following GO biological processes: blood vessel development (*RAPGEF1, HHIP, BCAS3, PKD1*), circulatory system development (*RAPGEF1, FGFRL1, HHIP, BCAS3, SGCD, PKD1*), muscle structure development (*FGFRL1, SGCD, COL6A3, ZFHX3, CEACAM5*), epithelium development (*KLK14, RAPGEF1, EVPL, HHIP, PLOD1, PKD1*), epidermis development (*KLK14, EVPL, PLOD1*), regulation of cell junction assembly (*RAPGEF1, BCAS3*), biological/cell adhesion (*RAPGEF1, FGFRL1, BCAS3, COL6A3, CEACAM5, PKD1*), detection of stimulus (*OR4C3, PKD1*), response to endogenous stimulus (*RAPGEF1, FGFRL1, ZFHX3, HHIP, PLOD1, BCAS3, ALPP*), regulation of cytokine biosynthetic process (*MAP2K3, TLR8*), response to growth factor (*RAPGEF1, FGFRL1, ZFHX3, HHIP, ALPP*), postreplication repair/protein K63‐linked ubiquitination (*UBE2NL*), and protein‐containing complex assembly (*PDE4DIP, FGFRL1*). Regulation of transcription DNA‐templated (*ZNF717*) was identified in GeneCards platform (Stelzer et al., 2016). Moreover, no data were available within GO biological process for *SPATA17* and *CFAP65* genes; however, GO molecular terms were related to calmodulin and RNA binding, respectively (Stelzer et al., 2016).

## 4 DISCUSSION

The present article is the first study of a role for genes and genetic variants on familial recurrence of gastroschisis. NGS application using a family‐based model system was a useful modality to identify DNA variants with a likely gene‐disrupting effect segregating with gastroschisis using WES. The two cases on this family could suggest a monogenic origin of gastroschisis; however, no single gene‐disrupting variant was identified. With a subsequent analysis, we identified a set of rare and low‐frequency coding nonsynonymous variants predicted with potential deleterious effects that could be potential candidates for future studies (Tables 1 and 2).

### 4.1 A multifactorial model in gastroschisis

The pursuit of the genetic variants that provide integration of an accurate genetic counseling or individual disease prediction to develop gastroschisis is challenging (Salinas‐Torres et al., 2018b). In the present study, we focused on two affected half‐sisters with a confirmed isolated anomaly. Further clinical features related to these cases included young maternal age with a low prepregnancy body mass index, limited preconceptional care, genitourinary infections, change in paternity, as well as potential exposures such as parental alcohol intake and cigarette smoking. Therefore, our findings suggest that a complex interplay between genetic and nongenetic factors may be precipitating elements in the development of gastroschisis (Salinas‐Torres et al., 2018; Salinas‐Torres et al., 2018b,c,d).

With certainty, the underlying mechanism for the defect seems complex and involves several functionally interacting genes for which all possible genetic and nongenetic factors contributing to complex landscape should be considered. In this sense, the multiple factors and gene interactions identified, particularly, background modifiers (Salinas‐Torres et al., 2019a,b), can aggravate or mask the expected phenotype through gain‐ or loss‐of‐function mutations in genes involved in related cellular functions (Hou, Leeuwen, Andrews, & Boone, 2018). Accordingly, the genetics of complex traits is significant, considering that even minor fluctuations in a modest number of genes or gene pathways with disease‐relevant cells, as well as their direct regulators, may contribute to disease susceptibility, as depicted in the “omnigenic” hypothesis (Boyle, Li, & Pritchard, 2017). Thus, considering our results from gene prioritization based on significant phenotypic relevance and functional properties, multiple defects in biological processes and pathways causing cumulative effects could be orchestrated by a “multifactorial model” probably leading to susceptibility or phenotypic variability in gastroschisis (Chakravorty & Hegde, 2018; Salinas‐Torres, Gallardo‐Blanco, Salinas‐Torres, Cerda‐Flores, et al., 2019; Salinas‐Torres et al., 2018; Salinas‐Torres et al., 2018b).

The above highlights several of the limitations of this study. First, in the absence of functional validation, findings from variant prediction algorithms can only be interpreted as suggestive evidence. Second, lacking assessment of the role of other types of genetic variation, such as structural or copy number variants, was minimized because criteria for WES evaluation were based on a family without evidence of multiple congenital anomalies or a syndrome. Third, given that no single gene‐disrupting variant was identified neither in the trio‐based WES nor in the affected half‐sister WES suggesting a monogenic influence, a multifactorial approach was considered in spite of limited investigation regarding gene–gene or gene–environment interactions in gastroschisis susceptibility. Finally, target samples validated from two different sequencing technologies could also affect our findings. WES depth coverage at 10x was 98.60% and 97.26% by Nextera and Agilent technologies, respectively, which agrees with a previous study indicating no significant difference in coverage efficiency between these technologies and thereby, giving a high technical reproducibility (Chilamakuri et al., 2014).

### 4.2 Novel candidate genes for gastroschisis

Given the current paucity of knowledge regarding genes in humans that influence normal development or significant causal implication in gastroschisis, a direct role in the defect from genes and gene variants identified by WES is unfamiliar. Nevertheless, significant genetic influences displaying plausibility within biological pathways and functional GO categories possibly related to gastroschisis were identified (Table S1).

Overall, these candidate genes highlight a role for the development of blood vessel, circulatory system, muscle structure, epithelium, and epidermis, as well as regulation of cell junction assembly, biological/cell adhesion, detection of stimulus, response to endogenous stimulus, regulation of cytokine biosynthetic process, response to growth factor, postreplication repair/protein K63‐linked ubiquitination, protein‐containing complex assembly, and regulation of transcription DNA‐templated in gastroschisis pathogenesis. Thus, our findings point to a complex interplay of functionally interacting genetic and nongenetic factors, thereby each of these interacting genes and genetic variations are likely substantial influences to the development of gastroschisis, underscoring major gaps concerning the current knowledge of the genetics and developmental biology of the defect.

In conclusion, we identified novel gastroschisis susceptibility genes using WES from a Mexican family with recurrence of the defect. These candidate genes were involved in several crucial GO biological processes possibly underlying gastroschisis. We, therefore, hypothesize that an orchestrated genetic cascade of morphogenetic events must occur, in order to impact normal ventral body wall closure as well as gastroschisis developmental process (Salinas‐Torres, Gallardo‐Blanco, Salinas‐Torres, Cerda‐Flores, et al., 2019). Thus, considering the likely gene‐disrupting effect prediction results and similar biological pattern of mechanisms, we propose a joint “multifactorial model” for this family and perhaps, gastroschisis developmental process.

## ETHICAL APPROVAL AND INFORMED CONSENT

The present study was approved by the Institutional Ethics Committee from the School of Medicine and University Hospital “Dr. José Eleuterio González”, Universidad Autónoma de Nuevo León, México (Approval: GN17‐00002). Written informed consents were obtained from the parents.

## CONFLICT OF INTEREST

The authors declared no potential conflict of interest and received no financial support concerning the research, authorship, or publication of this article.
