# Supplementary material for: Whole exome sequencing identifies multiple novel candidate genes in familial gastroschisis
Source: Mol Genet Genomic Med. 2020 Mar 12;8(5):e1176. doi: 10.1002/mgg3.1176 (PMC7216806; doi:10.1002/mgg3.1176)
Supplement: Supplementary file 1 [file MGG3-8-e1176-s001.pdf]

Supplemental Table 1. Functional GO categories, function, and disorders associated with novel candidates genes in gastroschisis through whole exome sequencing

Gene	Chromosome position (hg19)	Top functional categories (pathways//GO biological process)†	Function//disorders associated†
SPATA17	chr1-217804722	—//—	Calmodulin binding// Spermatogenic failure X-linked 1, autosomal recessive congenital ichthyosis
PDE4DIP	chr1-144852390	—//Cellular protein-containing complex assembly, regulation of Golgi organization	Participates in microtubule dynamics promoting microtubule assembly//Pineoblastoma, cardiomyopathy
CFAP65	chr2-219903258	—//—	May play role in sperm motility//—
ALPP	chr2-233245141	Vesicle-mediated transport, folate biosynthesis, metabolism, thiamine metabolism, embryonic and induced pluripotent stem cell differentiation pathways and lineage-specific markers//Dephosphorylation	Metalloenzyme that catalyzes the hydrolysis of phosphoric acid monoesters//Obstructive jaundice, hypophosphatasia, hepatic tuberculosis, seminoma
ZNF717	chr3-75790838; chr3-75788028	Gene expression, herpes simplex virus 1 infection//Regulation of transcription DNA-templated, regulation of transcription by RNA polymerase II	May be involved in transcriptional regulation//—
OR4C3	chr11-48347014	Signaling by GPCR, olfactory transduction//Signal transduction, G protein-coupled receptor signaling pathway, sensory perception of smell, response to stimulus	Odorant receptor that initiate a neuronal response that triggers the perception of a smell//—
MAP2K3	chr17-21204210	Activated TLR4 signalling, IL-2 pathway, 4-1BB pathway, toll-like receptor signaling pathway, CNTF signaling//Activation of MAPK activity, protein phosphorylation, inflammatory response, signal transduction, phosphorylation	Dual specificity kinase activated by cytokines and environmental stress in vivo. Catalyzes the concomitant phosphorylation of a threonine and a tyrosine residue in the MAP kinase p38//Chlamydia pneumonia, inhalation anthrax, cerebral cavernous malformations
TLR8	chrX-12924826	Activated TLR4 signalling, toll-like receptor signaling pathway, Th1 differentiation pathway, NF-KappaB family pathway, TRAF pathway// Microglial cell activation, regulation of protein phosphorylation, immune system process, MyD88-dependent toll-like receptor signaling pathway	Key component of innate and adaptive immunity//Superficial basal cell carcinoma, retinitis pigmentosa, trench fever, toxic pneumonitis, inflammatory bowel disease
UBE2NL	chrX-142967468	Ubiquitin mediated proteolysis, protein ubiquitylation// Postreplication repair, protein K63-linked ubiquitination	Ubiquitin conjugating enzyme activity//—

Gene	Chromosome position (hg19)	Top functional categories (pathways//GO biological process)†	Function//disorders associated†
PLOD1	chr1-12025529	Collagen chain trimerization, lysine degradation, degradation of the extracellular matrix//Response to hypoxia, cellular protein modification process, epidermis development, peptidyl-lysine hydroxylation	Catalyzes hydroxylation of lysine residues in collagen alpha chains and is required for normal assembly and cross-linkling of collagen fibrils//Ehlers-danlos syndrome kyphoscoliotic type 1, umbilical hernia, omphalocele
COL6A3	chr2-238256472	Collagen chain trimerization, integrin pathway, ERK signaling, PI3K-Akt signaling pathway, phospholipase-C pathway//Growth plate cartilage chondrocyte morphogenesis, cell adhesion, muscle organ development, negative regulation of peptidase activity, negative regulation of endopeptidase activity	Acts as a cell-binding protein// Dystonia 27, Bethlem myopathy 1, Ullrich congenital muscular dystrophy 1, muscular disease
FGFRL1	chr4-1018891	Negative regulation of FGFR1 signaling, VEGF signaling pathway, MAPK signaling: mitogen stimulation pathway, mTOR signaling pathway, signaling by GPCR//Skeletal system development, heart valve development, negative regulation of cell proliferation, fibroblast growth factor receptor signaling pathway	Negative effect on cell proliferation//Wolf-Hirschhorn syndrome, vesicoureteral reflux 1
HHIP	chr4-145659050	Hedgehog signaling pathway, Wnt signaling pathways: beta-catenin-dependent Wnt signaling, cAMP signaling pathway//Signal transduction, neuroblast proliferation, animal organ morphogenesis, dorsal/ventral pattern formation, negative regulation of signal transduction	Modulates hedgehog signaling in several cell types//Stature quantitative trait locus 12, chondrodysplasia Blomstrand type, metaphyseal chondrodysplasia Jansen type, basal cell carcinoma, holoprosencephaly
SGCD	chr5-156022053	Arrhythmogenic right ventricular cardiomyopathy, dilated cardiomyopathy, allograft rejection// muscle organ development, cell death, calcium-mediated signaling, cellular protein-containing complex localization, cardiac muscle tissue development	Forms a link between the F-actin cytoskeleton and the extracellular matrix//Limb-girdle muscular dystrophy, familial isolated dilated cardiomyopathy, dilated cardiomyopathy

Gene	Chromosome position (hg19)	Top functional categories (pathways//GO biological process)†	Function//disorders associated†
RAPGEF1	chr9-134501557	RET signaling, development HGF signaling pathway, focal adhesion, MET promotes cell motility, common cytokine receptor gamma-chain family signaling pathways//Activation of MAPKK activity, blood vessel development, signal transduction, transmembrane receptor protein tyrosine kinase signaling pathway, small GTPase mediated signal transduction	Guanine nucleotide-releasing protein that binds to SH3 domain of CRK and GRB2/ASH. Transduces signals from CRK to activate RAS. Plays a role in the establishment of basal endothelial barrier function//Hemolytic uremic syndrome atypical 1
PKD1	chr16-2153528	Cargo trafficking to the periciliary membrane, organelle biogenesis and maintenance, simplified interaction map between LOXL4 and oxidative stress pathway//Cartilage condensation, blood vessel development, in utero embryonic development, kidney and liver development	Regulator of calcium permeable cation channels and intracellular calcium homoeostasis. It is also involved in cell-cell/matrix interactions//Polycystic kidney disease 1 with or without polycystic liver disease, end stage renal failure, multicystic dysplastic kidney
ZFHX3	chr16-72821344	Signaling pathways regulating pluripotency of stem cells, transcriptional regulatory network in embryonic stem cell, circadian rythm related genes//Negative regulation of transcription by RNA polymerase II, regulation of transcription DNA-templated, transcription by RNA polymerase II, cell cycle arrest, brain development	Transcriptional regulator which can act as an activator or a repressor. Inhibits the enhancer element of the AFP gene by binding to its AT-rich core sequence. In concert with SMAD-dependent TGF-beta signaling can repress the transcription of AFP via its interaction with SMAD2/3. Regulates the circadian locomotor rhythms via transcriptional activation of neuropeptidergic genes which are essential for intercellular synchrony and rhythm amplitude in the suprachiasmatic nucleus of the brain//Prostate cancer, atrial fibrillation, shoulder impingement syndrome
BCAS3	chr17-59152328	Ectoderm differentiation//Angiogenesis, Golgi organization, positive regulation of endothelial cell migration, microtubule organizing center organization, negative regulation of GTPase activity	Plays a role in angiogenesis. Participates in the regulation of cell polarity and directional endothelial cell migration by mediating both the activation and recruitment of CDC42 and the reorganization of the actin cytoskeleton at the cell leading edge. Functions synergistically with PELP1 as a transcriptional coactivator of estrogen receptor-responsive genes. Stimulates histone acetyltransferase activity. Binds to chromatin//Breast cancer

Gene	Chromosome position (hg19)	Top functional categories (pathways//GO biological process)†	Function//disorders associated†
EVPL	chr17-74019638	Keratinization, developmental biology, cytoskeleton remodeling neurofilaments//Epidermis development, peptide cross-linking, keratinocyte differentiation, wound healing	Component of the cornified envelope of keratinocytes. May link the cornified envelope to desmosomes and intermediate filaments//Paraneoplastic pemphigus, pemphigus foliaceus, familial pemphigus vulgaris, bullous skin disease
KLK14	chr19-51582808	Collagen chain trimerization, keratinization, MSP-RON signaling, developmental biology//Proteolysis, fertilization, negative regulation of G protein-coupled receptor signaling pathway, positive regulation of G protein-coupled receptor signaling pathway, epidermis morphogenesis	Serine-type endopeptidase with a dual trypsin-like and chymotrypsin-like substrate specificity. May function through desmoglein DSG1 cleavage in epidermal desquamation a process by which the most superficial corneocytes are shed from the skin surface. May be involved in several aspects of tumor progression including growth, invasion and angiogenesis//—
CEACAM5	chr19-42225021	Metabolism of proteins, adhesion, NF-kappaB signaling, hematopoietic stem cell differentiation pathways and lineage-specific markers, cell surface interactions at the vascular wall//Apoptotic process, cell adhesion, homophilic and heterophilic cell adhesion via plasma membrane adhesion molecules, negative regulation of anoikis	Cell surface glycoprotein that plays a role in cell adhesion, intracellular signaling and tumor progression. Plays a role as an oncogene by promoting tumor progression; induces resistance to anoikis of colorectal carcinoma//Lung cancer, colorectal cancer, papillary adenocarcinoma, gastrointestinal carcinoma

†Functional annotation is shown according to GeneCards SuperPathways and Gene Ontology top biological process categories, whereas function and disorders associated with genes were described from GeneCards platform (Stelzer et al., 2016).
